# Fresh litter acts as a substantial phosphorus source of plant species appearing in primary succession on volcanic ash soil

**DOI:** 10.1038/s41598-021-91078-6

**Published:** 2021-06-01

**Authors:** Sae Katayama, Takayuki Omori, Masaki Tateno

**Affiliations:** 1 Nikko Botanical Garden, Department of Biological Sciences, Graduate School of Science, The University of Tokyo, Tochigi, Japan; 2 Laboratory of Radiocarbon Dating, The University of Museum, The University of Tokyo, Tokyo, Japan

**Keywords:** Ecology, Plant sciences

## Abstract

Plants have difficulty absorbing phosphorus from volcanic ash soils owing to the adsorption of phosphorus by aluminum and iron in the soils. Thus, on volcanic ash soils, the phosphorus source for natural vegetation is expected to be organic matter, however, there is a lack of experimental evidence regarding this occurrence. Here, we studied the effect of organic matter on plant growth of some species that occur in primary successions of volcanic ash soil ecosystems, based on growth experiments and chemical analyses. We found that a large amount of inorganic phosphorus (but only a limited amount of inorganic nitrogen) is leached from fresh leaf litter of the pioneer spices *Fallopia japonica* at the initial stage of litter decomposition. Phosphorus from the fresh litter specifically activated the growth of subsequently invading nitrogen-fixing alder when immature volcanic soil was used for cultivation. In contrast, old organic matter in mature soil was merely a minor source of phosphorus. These results suggest that fresh litter of *F. japonica* is essential for growth of nitrogen-fixing alder because the litter supplies phosphorus. We consider that rapid phosphorus cycles in fresh litter-plant systems underlie the productivity of natural vegetation even in mature ecosystems established on volcanic ash soils.

## Introduction

The availability of phosphorus, one of the major nutrients essential for plant growth, is determined by biological and geochemical sources^1^, and can be limiting in most of the natural environment^2^, especially in volcanic ash soils. A model of long-term phosphorus dynamics in volcanic ash soil has previously been reported^3^. Phosphorus is first contained in calcium phosphate as apatite; however, as weathering progresses, Al and Fe strongly absorb the phosphorus within the volcanic ash soil making it largely insoluble.

Previous studies have reported that some plants can release organic acids that transform the phosphorus in volcanic ash soil into a usable form. For example, Lupine releases such organic acids from its roots, and can grow in soils with low phosphorus availability^4^. Leguminous crops, also known to release organic acids, have likewise been reported to absorb phosphorus from volcanic ash soil^5,6^. When iron phosphate or aluminum phosphate in soil reacts with organic acids released by plants, a metal complex is formed, and phosphorus is solubilized as a result^7^. In addition, it has been reported that root-associated fungi contribute to plant growth in phosphorus-limitation conditions^8,9^.

Organic matter, such as litter, is thought to be a source of phosphorus. Some studies have suggested that phosphorus easily leaches from litter on Hawaiian volcanoes^10,11^, suggesting that soil organic matter, including litters and old humus, supply phosphorus to vegetation. Other studies suggested that organic phosphorus is one of the most important fractions in predicting phosphatase activity^12^. Thus, the biological cycle of phosphorus in forests has been an important subject and quantitative evaluation of each process is still currently under development.

Despite volcanic ash soil covering about 20% of the land in Japan^13^,and phosphorus deficiency being a serious problem in Japanese agriculture^14^, net primary production in Japanese forests is primarily is not low compared to other temperate zones of the world^15–18^. This suggests that natural vegetation on the infertile volcanic ash soil obtain sufficient nutrition including phosphorus.

Here we investigate how plants that grow in primary succession obtain phosphorus in volcanic ash soil, which has a strong phosphorus adsorption capacity. This study aimed to clarify whether fresh litter and old organic matter deposited on the soil surface was a phosphorus source for natural vegetation on volcanic ash soils and whether they contributed to vegetation development. We focused on organic matter and set up a working hypothesis that phosphorus leached from the organic matter promotes plant growth in volcanic ash soil. First, growth experiments and chemical analyses were conducted to determine whether phosphorus leached from fresh litter promoted plant growth in a typical primary succession plant species growing in volcanic soils—*Fallopia japonica*, *Alnus inokumae*, and *Betula ermanii.* They were grown in immature volcanic ash soil and their growth rate, with and without litter, were compared. Second, nutrient supply by old organic matter in mature volcanic ash soil was verified similarly as described above. These experiments show that fresh litter leaves are a major source of natural vegetation in volcanic ash soil and that old organic matter in mature soil is merely a minor phosphorus source. Finally, we discussed the possibility of a rapid phosphorus cycle in fresh litter-plant systems that underlie the productivity of natural vegetation established on volcanic ash soil.

## Materials and methods

### Volcanic ash soil

We used commercially available Kanuma soil as volcanic ash soil (fine-grained pumice, Akagi Engei Co., Ltd.) for growth experiment 1. Kanuma soil is a fully weathered pyroclastic fall from the eruption of Mt. Akagi 44,000 years ago^19^. The soil contains 30.8% aluminum (allophane and imogolite) and 1.4% iron (ferrihydrite)^20^. For growth experiment 2, we used three natural volcanic ash soil types—immature soil of pumice (Kanuma soil, C horizon), as well as mature soils of andosol (A–B horizon) and topsoil (the surface of andosol, P to A horizon)—collected from a riverbed in Kanuma City (36°35′ N, 139°44′ E; 200 m a.s.l.), central Japan, where the vegetation is a cypress forest. This place is managed by the Kanuma Civil Engineering Office. The topsoil was collected at a depth of approximately 0–10 cm from the soil surface after removing the fallen leaves on the soil surface. The andosol layer, typically distributed at a depth of approximately 10–75 cm, was collected from a depth of approximately 10–30 cm. Below the andosol layer, the Akadama soil layer is distributed; further below, the pumice Kanuma soil is distributed. The pumice was collected approximately 50 cm under the Akadama layer.

Temporal information on soil formation was confirmed by direct radiocarbon dating of the soil samples. After removing soil carbonate with 1.0 M HCl, the total organic fraction was analyzed using an accelerator mass spectrometer (0.5MV compact AMS system, NEC) at the laboratory of radiocarbon dating, University of Tokyo. Conventional radiocarbon age after correction of isotopic fractionation with δ^13^C values was calibrated to a calendar date with the calibration dataset IntCal13^21^.

The elemental analysis of total phosphorus, nitrogen, and carbon in the soil samples was performed by Createrra Inc. (http://www.createrra.co.jp/english/top.html).

### Plant species

On the volcanic ash soil of Mt. Fuji, Japan's highest volcano, vegetation in primary succession generally changes from herbaceous plants such as *Fallopia japonica* (Houtt.) Ronse Decr. var. japonica to nitrogen-fixing alder plants, and finally to non-nitrogen fixing *Betula ermanii* Cham^22,23^. Hence, we used three species—*F. japonica,* the alder species *Alnus inokumae* Murai et Kusaka, and *B. ermanii*—owned by and grown in our research institute, Nikko Botanical Garden, for the growth experiments. Experimental research on these plants, including the collection of plant material, comply with the relevant institutional, national, and international guidelines and legislation.

### Litter incubation experiment

Samples (1 g) of *F. japonica* litter leaves—collected upon leaf fall on an autumn day, dried at 80 °C for at least 48 h, and then crushed—were placed in cultivating tubes (*n* = 5). Then, 5 g of wet soil from the Nikko Botanical Garden (36°45′ N, 139°35′ E; 647 m a.s.l.) in Nikko, central Japan, was added to 500 mL of water and stirred (solution I). As inoculation, 0.1 mL of the supernatant of solution I was added to the tubes^24^. Considering that the amounts of phosphorus and nitrogen in the solution I were approximately 0.003 mg/L and 0.3 mg/L, respectively, they were determined to have not affected the initial value (t = 0). Next, 2 mL of water was added to the tubes, which were then kept at 30 °C. The tubes were left open to maintain an aerobic environment. The efflux of phosphorus and nitrogen from the leaves was measured every week for ten weeks. For these measurements, 5 mL of water was added and the tube was centrifuged for 10 min (solution II). The supernatant of solution II was then used for phosphorus and nitrogen measurements, and the residue was continuously kept at 30 °C.

### Growth experiments

Growth experiments were conducted in an open-type greenhouse in Nikko Botanical Garden. The greenhouse is only vinyl on the ceiling and good ventilation to keep the temperature constant. The mean monthly highest and lowest temperatures and the monthly precipitation observed in the botanical garden during the cultivation period are provided in Table 1. In the growth experiments, irrigation with tap water was provided to the plants and litter leaves in the morning and evening. The phosphorus and nitrogen concentration of the tap water were approximately 0.03 mg/L and 0.25 mg/L respectively.

**Table 1:** Nikko botanical garden weather data (May–October 2019).

	May	June	July	August	September	October
Mean monthly highest temperature (°C)	21.7	21.5	23.8	28.4	24.6	19.1
Mean monthly lowest temperature (°C)	9.8	13.0	17.6	19.8	15.9	11.3
Monthly precipitation (mm)	187	332	298	324	137	650

#### Growth experiment 1: Comparative experiment on the growth of plant species with and without litter

The seedlings used for the experiment were from the species *F. japonica*, *A. inokumae*, and *B. ermanii*. A similar seedling size was used for each plant species. Seedlings of *A. inokumae* coexist with N-fixing actinomycetes.

Six plants per species were collected before cultivation (t = 0) and dried in an oven at 80 °C for at least 48 h to measure the dry weight. There were four experimental groups for each species: a control (Con), a nitrogen addition (N: 10 mM NH_4_NO_3_), a phosphorus addition (P: 10 mM NaH_2_PO_4_), and a nitrogen and phosphorus addition (NP: 10 mM NH_4_NO_3_ + 10 mM NaH_2_PO_4_). Once a week, 50 mL of each nutrients was added to a 0.25-L garden pot. To verify whether the addition of litter (denoted by +) improved plant growth, litter leaves of *F. japonica* were placed on the soils. To verify if nutrients leached from litter sustained plant growth, we also combined nutrient and litter additions (Con+, N+, P+, NP+). When nutrients were added to the soil once a week, litter bag was removed before fertilizer application and returned after that.

To reproduce how litter is deposited and supplies nutrients on volcanic ash soil in primary succession, *F. japonica* litter was collected in Nikko in the autumn of 2018 and dried at 80 °C or 2 days or more (the same litter was used in incubation). Approximately 9 g of litter leaves was packed in a tea mesh bag^25^ to prevent it from flying in the wind and placed on the soil surface of the garden pots. As indicated by the equation below, the amount of litter added to the 8 × 8 cm (0.0064 m^2^) garden pot used in this experiment amounts to approximately three years of litter production when converted to the amount of leaf litter in a 15-year-old alder forest, i.e., about 430 g/m^2^ per year^26^.

$$\frac{9\,g}{{430\frac{g}{{ m^{2} }} yr \times 0.0064 m^{2} }} \cong 3.3 yr$$

Six seedlings per group of *A. inokumae* and *B. ermanii* were cultivated for approximately 2 months (June 7–August 22, 2019) and 12 seedlings per group of *F. japonica* were cultivated for about 1 month (September 10–October 15, 2019). The experiment was stopped after 1 month for *F. japonica* as it grew rapidly in 2 nutrient conditions (NP, NP+) and the roots overflowed from the garden pot. At the end of the experiment, growth was evaluated by measuring dry weight after drying seedlings at 80 °C for at least 48 h. Subsequently, the total phosphorus and nitrogen content of the dried seedlings were also measured (chemical analysis).

The mass of phosphorus leached from litter during the cultivation period was calculated from the difference in the phosphorus contents of the litter before and after cultivation.

#### Growth experiment 2: Comparative experiment on plant growth with old organic matter

Eight *F. japonica* seedlings were cultivated in three different soil-types (pumice, andosol, and topsoil, as mentioned above) under three experimental conditions (Con, N, P, same nutrition as growth experiment 1) from May 29 to July 12, 2019. These plants were then harvested and oven-dried at 80 °C for at least 48 h to measure dry weight. Subsequently, the total phosphorus and nitrogen content of the seedlings were also measured (chemical analysis).

### Chemical analysis

#### Phosphorus

We used the dry destruction method to pretreat total phosphorus measurements in plant tissue^27^. A sample of the plant (0.05 g) was burned at 550 °C for 1 h. The plant ash was dissolved in 10 mL of 2 M H_2_SO_4_ and shaken for over 16 h; then, the solution was filtered. The filtrate was diluted at a 1:10 ratio with tris(hydroxymethyl)aminomethane (pH 8.0).

The soil for available phosphorus were pretreated by Truog’ s method^28^. The soil (0.05 g) was dissolved in 10 mL of 0.002 M H_2_SO_4_, shaken for 30 min, and the solution was filtered. The filtrate was diluted at a 1:10 ratio with water.

The amount of phosphorus in the sample solution was measured by the molybdenum blue colorimetric method^29^.

#### Nitrogen

The total nitrogen in plant tissue was measured using an elemental analyzer (EA; Vario Macro cube, Elementar, Germany). A few milligrams of the dried plant sample were placed in a tin capsule for EA combustion. EA carried out sample combustion and N_2_ separation/detection from the combusted gases and provided us with nitrogen contents.

The soil sample preparation for available nitrogen measurements was based on the incubation methodology^30^. Half of the sampled soils were analyzed fresh, and the other half incubated for four weeks at 30 °C before analysis. 2 M KCl (20 mL) was added to 2 g of the soil sample; the solution was shaken for 1 h and filtered. The filtrate was collected, and the volume of nitrogen was measured by indophenol blue absorptiometry after reducing all to ammonia using Pack Test WAK-TNi (Kyoritsu Chemical-Check Lab., Corp, Tokyo, Japan). Available nitrogen was taken as the difference in the concentration of inorganic nitrogen (NO_3_-N, NO_2_-N and NH_4_-N) between incubated and fresh soil.

### Statistical analysis

All statistical analyses were performed with EZR^31^ (Saitama Medical Center, Jichi Medical University, Saitama, Japan), which is a graphical user interface for R (The R Foundation for Statistical Computing, Vienna, Austria). More precisely, it is a modified version of R commander designed to add statistical functions frequently used in biostatistics. The figure’s values are mean ± SE. Intergroup differences for nutrition conditions in soil, and soil-types were evaluated using non-parametric Kruskal–Wallis with post-hoc Steel–Dwass tests. In addition, comparisons between with or without litter were evaluated using two-tailed Mann–Whitney U-test. *p* values are * *p* < 0.05, ** *p* < 0.01, *** *p* < 0.001.

## Results

### Litter incubation experiment

**Figure 1:**
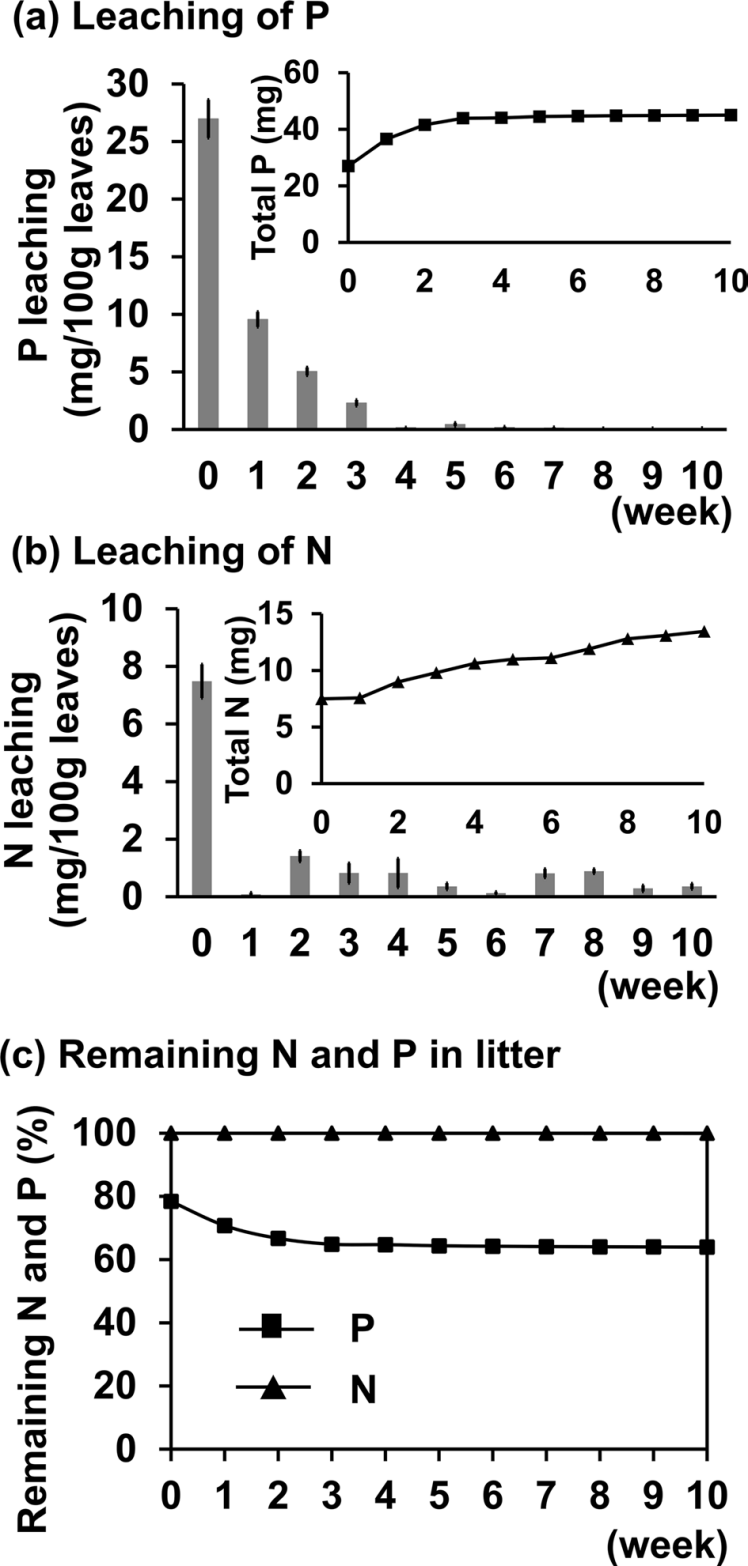
The amount of nitrogen (N) and phosphorus (P) leached from litter leaves per week. (**a**) Leaching of P from 100 g leaves (*n* = 5). *Inset* shows total P leached. (**b**) Leaching of N from 100 g leaves (*n* = 5). *Inset* shows total N leached. (**c**) Remaining N and P in litter leaves per week. The raw data are available in Supplementary Table S1. P was leached in large quantities from the beginning of organic matter decomposition.

Inorganic phosphorus and nitrogen liberated from the litter are shown in Fig. 1. About 20% of total phosphorus in litter leaves was inorganic phosphorus at t = 0 (80% was organic and remaining in litter), and about 40% of the total phosphorus was leached during the initial three weeks (Fig. 1c). In contrast, as compared to phosphorus, only small relative changes were observed in leached nitrogen.

### Growth experiment 1

**Figure 2:**
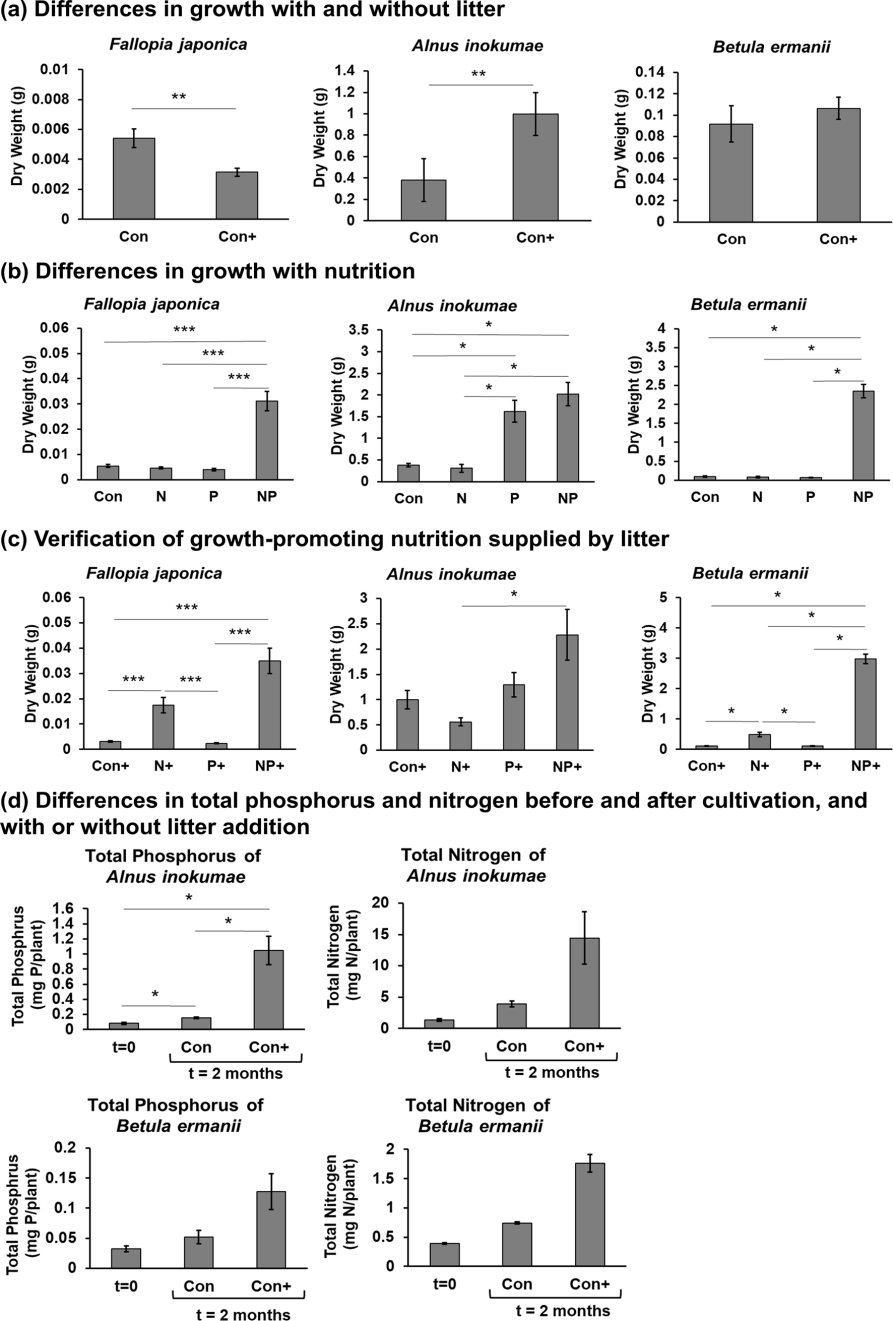
Effect of litter on the growth of *Fallopia japonica* (*n* = 11–12), *Alnus inokumae* (*n* = 5–6), and *Betula ermanii* (*n* = 5–6). (**a**) Differences in growth with and without litter. (**b**) Differences in growth with nutrition. (**c**) Verification of growth-promoting nutrition supplied by litter. (**d**) Differences in total phosphorus (*n* = 5–6) and nitrogen (*n* = 3) of *A. inokumae* and *B. ermanii* before and after cultivation, and with or without litter addition. The raw data are available in Supplementary Table S2. The figure’s value are mean ± SE. Mann–Whitney U-test was used for (**a**). Kruskal–Wallis with post-hoc Steel–Dwass tests were used for (**b**), (**c**) and (**d**). *p* values are *p < 0.05, **p < 0.01, ***p < 0.001. Growth of *A. inokumae* was promoted by phosphorus (P) or litter leaves (+). Growth of *F. japonica* and *B. ermanii* were promoted by both nitrogen and phosphorus (NP) and both nitrogen and litter leaves (N+). Litter leaves (+) increased phosphorus, especially in *A. inokumae*, and increased nitrogen levels in plants.

The growth of *A. inokumae* was significantly increased with litter compared to that without litter (*A. inokumae*: Con vs. Con+, *p* = 0.009) (Fig. 2a, Supplementary Figure S1).

Compared to the control group, the growth of *F. japonica* and *B. ermanii* significantly increased in the groups treated with nitrogen and phosphorus (*F. japonica*: Con vs. NP, *p* = 0.0003; *B. ermanii*: Con vs. NP, *p* = 0.02), as shown in Fig. 2b and Supplementary Figure S1. The growth of *A. inokumae* significantly increased in the groups supplemented with phosphorus (*A. inokumae*: Con vs. P, *p* = 0.04; Con vs. NP, *p* = 0.03). Therefore, the limiting factors of nutrition in Kanuma soil were nitrogen and phosphorus for *F. japonica* and *B. ermanii*, and phosphorus for *A. inokumae*.

To verify the effects of litter addition for growth, we cultivated plant species with litter and nutrients. The growth of *F. japonica* and *B. ermanii* was significantly increased by the addition of nitrogen and litter (*F. japonica*: Con+ vs. N+, *p* = 0.0002; *B. ermanii*: Con+ vs. N+, *p* = 0.03), and there was almost no difference in *A. inokumae* by addition of nutrients (Fig. 2c, Supplementary Figure S1). These results suggest that all plants would obtain a sufficient amount of phosphorus from litter.

Accumulation of phosphorus and nitrogen in plants with or without litter was shown in Fig. 2d. For *A. inokumae* and *B. ermanii*, the total phosphorus and total nitrogen in the plant were measured at the start of the growth experiment (t = 0) and after cultivation (t = 2 months). Since the mass *F. japonica* at t = 0 was insufficient for elemental analysis, *F. japonica* was not included in Fig. 2d. The addition of litter (Con+) increased the total phosphorus and nitrogen contents (Fig. 2d). Especially, total phosphorus contents of *A. inokumae* significantly increased by litter additions (*A. inokumae*: t = 0 vs. Con+, *p* = 0.01; Con vs. Con+, *p* = 0.02). During the cultivation period, phosphorus leached from litter was more than that absorbed by plants (Table 2). Therefore, litter is considered the major phosphorus source of the litter addition experiments, especially for *A. inokumae*.

**Table 2:** Amount of leached phosphorus during the cultivation period (*n* = 4).

Plant species	Cultivation period (months)	Total Phosphorus in litter (mg)				Amount of leached phosphorus during cultivation period	
		Before cultivation		After cultivation			
		Mean	SE	Mean	SE	(mg/pot)	(mg/m^2^)
*Alnus inokumae* *Betula ermanii*	2	11.2	0.2	6.4	1.2	4.8	751
*Fallopia japonica*	1	11.2	0.2	9.8	0.4	1.5	228

### Growth experiment 2

Growth experiment 1 was conducted to verify the effects of litter, the new organic matter. However, old organic matter is also present in mature natural vegetation volcanic ash soil; thus, we tried to verify the effects of old organic matter, using mature Kanuma soil collected from a cypress plantation.

Table 3 shows the age of organic matter in each soil. The organic fraction dates were 420 to 540 cal AD for the topsoil and 385 to 535 cal AD for the andosol (95.4% probability interval). The pumice could not be measured because of quite low carbon content.

**Table 3:** Soil conditions for pumice, andosol, and topsoil.

Soil type	Total carbon (g/kg)	Total Nitrogen (g/kg)	Total phosphorus (g/kg)	CN ratio			
**(A) Total element data**							
Topsoil	216	11.9	1.78	18			
Andosol	115	7.1	2.00	16			
Pumice	0.4	0.1	0.59	4			

Soil type	Available nitrogen (mg N/100 g of dry soil)					Available phosphorus (mg P/100 g of dry soil)	
	(1) 0 days		(2) 28 days		Available nitrogen (2)–(1)		
	Mean	SE	Mean	SE		Mean	SE
**(B) Available element data (*n*** **=** **3–4)**							
Topsoil	0.4	0.1	12.9	0.8	12.5	0.12	0.02
Andosol	0.6	0.2	9.7	1.1	9.1	0.11	0.03
Pumice	0.4	0.2	8.6	0.2	8.2	0.07	0.02

Soil type	Lab. Num	Radiocarbon age	δ^13^C	Calendar date (cal AD)			
		(BP)	(‰)	68.2% prob,	95.4% prob,		
**(C) Radiocarbon data**							
Topsoil	TKA-21535	1583 ± 20	− 22.5	420–535	420–540		
Andosol	TKA-21536	1622 ± 20	− 24.2	395–530	385–535		
Pumice	–	N.D	–	–	–		

**Figure 3:**
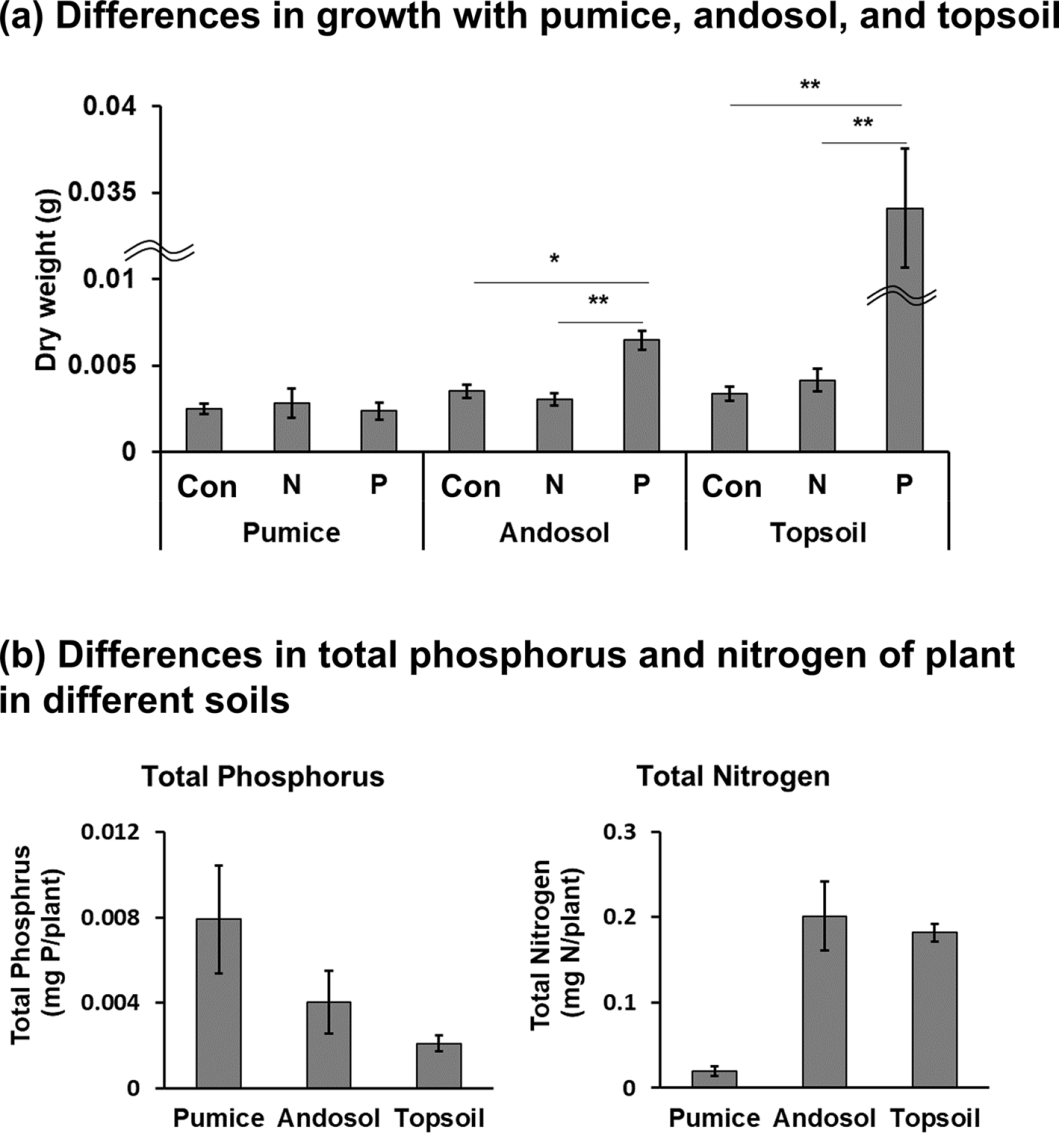
Verification of the effect of nutrients supplied from mature soil on plant growth. (**a**) Differences in growth of *Fallopia japonica* with pumice, andosol, and topsoil (*n* = 4–8). (**b**) Differences in total phosphorus (*n* = 4) and nitrogen (*n* = 3) of *F. japonica* in different soils. The raw data are available in Supplementary Table S3. The figure’s value are mean ± SE. Kruskal–Wallis with post-hoc Steel–Dwass tests were used for (**a**) and (**b**). *p* values are *p < 0.05, **p < 0.01. Phosphorus promoted *F. japonica* growth in andosol and topsoil. Total nitrogen was increased in plants cultivated in andosol and topsoil compared to that in pumice.

Phosphorus significantly promoted *F. japonica* growth in andosol and topsoil (andosol: Con vs. P, *p* = 0.01; topsoil: Con vs. P, *p* = 0.003) (Fig. 3a and Supplementary Figure S2). Figure 3b shows the total phosphorus and nitrogen contents of the plants after cultivation under control condition (Con), suggesting phosphorus deficiency in andosol and topsoil (Fig. 3b and Supplementary Table S3). Further, the plants grown on andosol and topsoil had considerably higher nitrogen contents than those grown on pumice, suggesting the existence of available nitrogen in andosol and topsoil (Table 3). These results indicate that the plants are able to absorb sufficient nitrogen from the mature soil containing old organic matter.

## Discussion

### Phosphorus leaching from litter promotes growth of N2-fixing *A. inokumae*, a succeeding species in the middle stage of primary succession

In the incubation experiment, approximately 40% of the total phosphorus of litter was in an inorganic form within three weeks after litter decomposition started (Fig. 1c). In contrast, almost all nitrogen persisted in an organic form incorporated in litter. These results suggest that a large amount of phosphorus can be supplied to plants from newly fallen litter, whereas little nitrogen is available. Tateno (1988) showed a similar tendency for nitrogen mineralization in an incubation experiment where nitrogen was not mineralized from fresh leaves of *F. japonica* in a 250 d incubation period, supporting our present result regarding nitrogen behavior in litter^24^.

In the growth experiment using immature pumice, the growth of N2-fixing *A. inokumae* was promoted by adding phosphorus or litter (Fig. 2a,b,c), while non-N2-fixing *F. japonica* and *B. ermanii* needed nitrogen in addition to phosphorus or litter. This is probably because the *Alnus* species coexist with N2-fixing actinomycetes^32^. In fact, *A. inokumae* had higher nitrogen content than that exhibited by *B. ermanii* (Fig. 2d) when only litter was placed on the pumice. In the same experiment, the addition of litter increased the amount of phosphorus absorbed by plants (Fig. 2d). This answers our research question, and shows that litter acts as a substantial phosphorus source for plants, at least in the case of volcanic ash soils, although mineral soil may be a phosphorus source in some cases^33^.

Similarly, the addition of litter increased the amount of nitrogen absorbed by plants (Fig. 2d). However, there was no improvement in the growth of non-N2-fixing *B. ermanii* by the addition of both phosphorus and litter. It is probable that, since plant tissues require more nitrogen than phosphorus, the amount of nitrogen leached from fresh litter is not sufficient for plant growth at the stage of primary succession of volcanic ash soil with low litter deposition.

**Figure 4:**
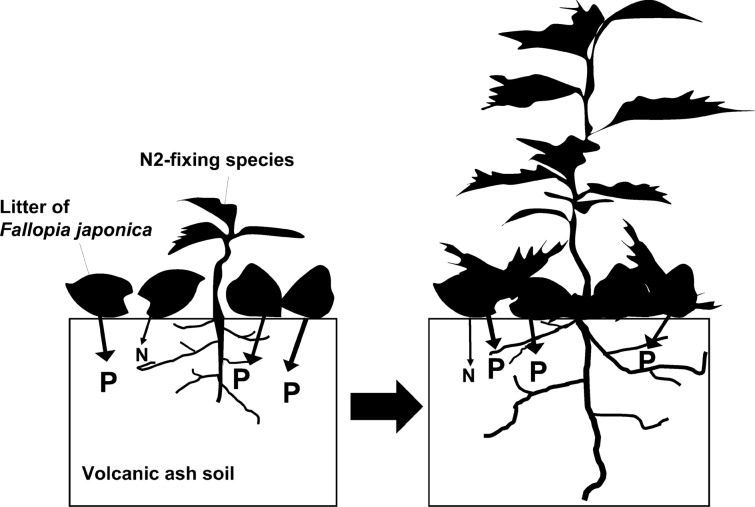
Relationship between litter and N2-fixing species. Alder with nitrogen-fixing abilities, invade during the middle of primary succession, gains phosphorus from new organic matter (e.g., fallen leaves of *Fallopia japonica*) on the soil surface, and promotes growth.

It is notable that even N2-fixing *A. inokumae* hardly grew on pumice without additional phosphorus or litter. In the middle stage of primary succession in Japanese volcanic ash soil, *A. inokumae* invades after *F. japonica*^23^. Therefore, *F. japonica* litter is essential for expressing the N2-fixing ability of *A. inokumae* because the litter supplies phosphorus to *A. inokumae* (Fig. 4).

Our results shows that litter acts as an important phosphorus source for plants. The results also give a cue for understanding N2 fixation in terrestrial ecosystems. N2-fixing species do not appear in early stage nor late stage of mature vegetation^22,23,34^. N2-fixing species cannot sufficiently absorb phosphorus without litter from pioneer species. Therefore, N2-fixing species cannot invade the early stage. Considering that N2 fixation is a costly process^35^, the advantage of N2-fixing ability disappears in the late stage of primary succession where N2-non-fixing species utilize sufficient nitrogen accumulated as a part of SOM.

### Rapid phosphorus cycle in ecosystems on volcanic ash soil

A question arises from our growth experiment: What is the nitrogen source in mature ecosystems in volcanic ash soil? Fig. 3a shows that the addition of phosphorus promotes the growth of *F. japonica* in andosol and topsoil of mature Kanuma soils, suggesting that phosphorus is the limiting nutrient in the soils and that plant species can absorb nitrogen in the soils. In fact, *F. japonica* grown on andosol and topsoil had considerably increased nitrogen contents compared to those grown on pumice (Fig. 3b). The topsoil and andosol contained large amounts of old organic matter (organic matter age: about 400 AD to 500 AD, Table 3). Therefore, we suggest that nitrogen can be supplied from the mature soil, while the major source of phosphorus is newly fallen litter, even in mature ecosystems established on volcanic ash soils. However, it is possible that the andosol and topsoil contained a small amount of new organic matter that could not be detected by radiocarbon dating, the age of the nitrogen source is still unknown.

These results suggest that old organic matter may act as a nitrogen source for plants. However, there is a problem remaining. Old organic matter in andosol and topsoil is presumed to be persistent humus formed from plant litter^36^. It is probable that the organic nitrogen component of humus^37^ decomposes over a long period of time. The difference in nitrogen mineralization rate between litter and humus may be explained by the CN ratio^38,39^. However, the CN ratio of *F. japonica* litter (Supplementary Table S4) was about 25 that was relatively similar to that of the humus in topsoil and andosol (Table 3). A similar tendency has previously been reported, which demonstrates that nitrogen hardly mineralizes after 250 d of litter and fresh leaves of *F. japonica* incubation, although the CN ratio of fresh leaves has been estimated to decrease to about 12 by the end of the incubation experiment^24^. Further research is required to investigate the mechanism of nitrogen mineralization in organic matter for nature of primary succession.

This study suggests that phosphorus is rapidly leached from litter and contributes to the vegetation development on volcanic ash soil. Model analysis of strongly weathered tropical soils deficient in phosphorus revealed that organic phosphorus was the major source of the available phosphorus^40^. The high productivity of tropical rainforests may be supported by the rapid leaching of phosphorus from fresh litter as shown in this study.

## Supplementary Information

Supplementary Information 1.

Supplementary Information 2.

Supplementary Caption.

## Data Availability

All data generated or analyzed during this study are included in this published article and its supplementary information files.
