# Supplementary material for: Fresh litter acts as a substantial phosphorus source of plant species appearing in primary succession on volcanic ash soil
Source: Sci Rep. 2021 Jun 1;11:11497. doi: 10.1038/s41598-021-91078-6 (PMC8169781; doi:10.1038/s41598-021-91078-6)
Supplement: Supplementary file 1 — Supplementary Information 1. [file 41598_2021_91078_MOESM1_ESM.pdf]

Fresh litter acts as a substantial phosphorus source of plant species appearing in primary succession on volcanic ash soil

Sae Katayama[1*], Takayuki Omori[2], and Masaki Tateno[1]

[1]Nikko Botanical Garden, Department of Biological Sciences, Graduate School of Science. The University of Tokyo, Nikko, Tochigi 321-1435, Japan

[2]The University of Museum, Laboratory of Radiocarbon Dating, the University of Tokyo, Hongo Tokyo, 113-0033, Japan

* Corresponding author

E-mail: sae-katayama@g.ecc.u-tokyo.ac.jp

Tel: +81-288-54-0206

Fax: +81-288-54-3178

ORCID iD: 0000-0002-7073-9199

Supplementary Information: Figure

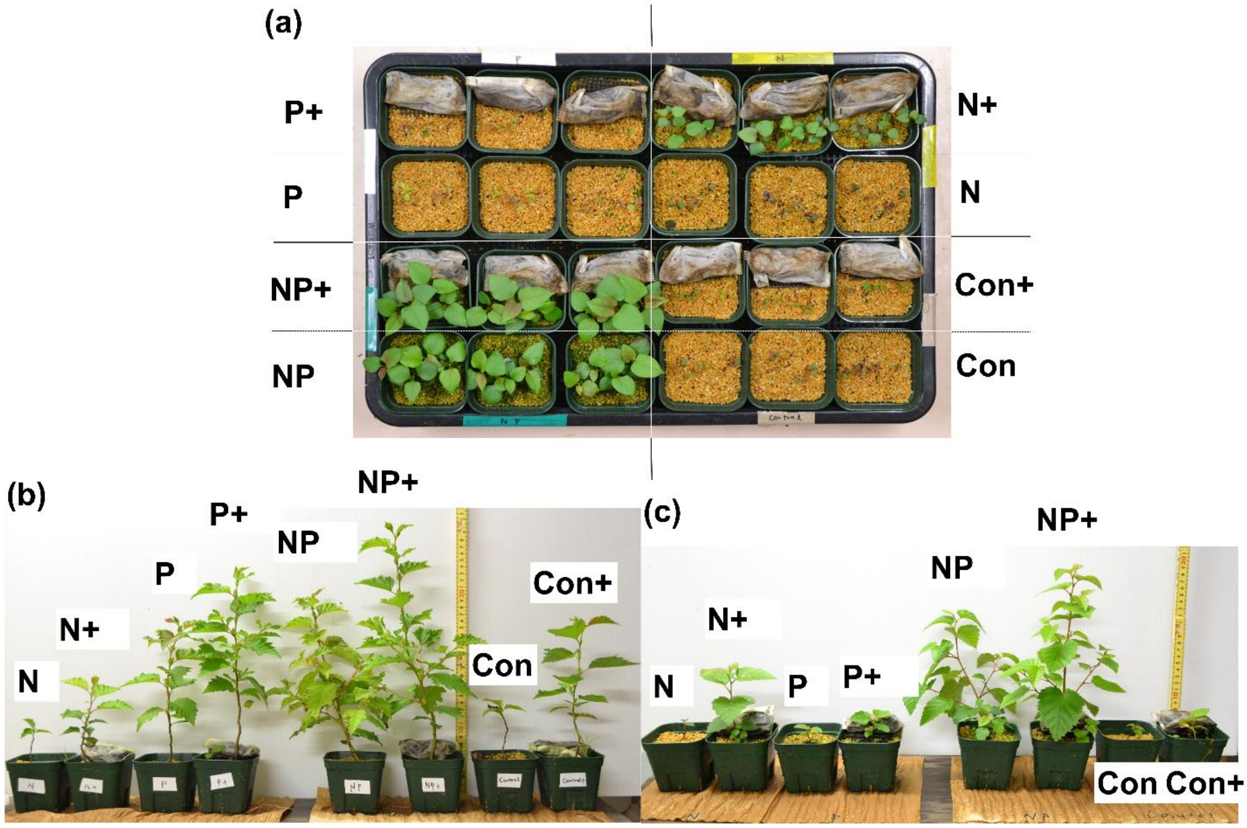

Figure S1. (a) *Fallopia japonica*, (b) *Alnus inokumae*, and (c) *Betula ermanii*, plants at the end of the cultivation experiments. Plants depicting standard growth were selected from each nutritional condition group. Photo credit: Sae Katayama. Con, control; +, added litter.

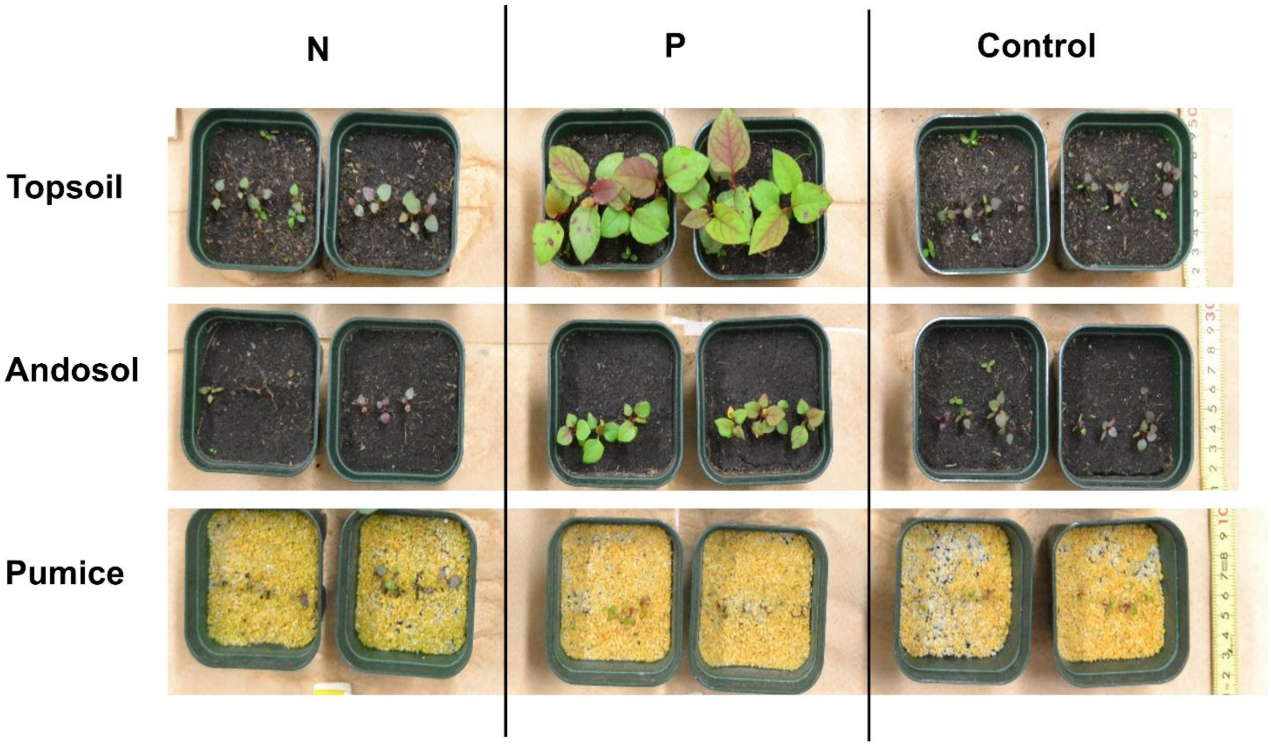

Figure S2. After plant cultivation of *Fallopia japonica* on pumice, andosol, and topsoil. Plants depicting standard growth were selected from each nutritional condition group. Photo credit: Sae Katayama.
