# Supplementary material for: Fresh litter acts as a substantial phosphorus source of plant species appearing in primary succession on volcanic ash soil
Source: Sci Rep. 2021 Jun 1;11:11497. doi: 10.1038/s41598-021-91078-6 (PMC8169781; doi:10.1038/s41598-021-91078-6)
Supplement: Supplementary file 3 — Supplementary Caption. [file 41598_2021_91078_MOESM3_ESM.docx]

**Table S1.** Raw data of litter incubation experiment

**Table S2.** Raw data of growth experiment 1

**Table S3.** Raw data of growth experiment 2

**Table S4.** Raw data of CN ratio of *Fallopia japonica* litter
